# Investigation of the caspase-dependent mitochondrial apoptotic pathway in mononuclear cells of patients with systemic lupus erythematosus

**DOI:** 10.1186/s12967-014-0303-1

**Published:** 2014-11-06

**Authors:** Yu-Jih Su, Tien-Tsai Cheng, Chung-Jen Chen, Wen-Neng Chang, Nai-Wen Tsai, Chia-Te Kung, Hung-Chen Wang, Wei-Che Lin, Chih-Cheng Huang, Ya-Ting Chang, Chih-Min Su, Yi-Fang Chiang, Ben-Chung Cheng, Yu-Jun Lin, Cheng-Hsien Lu

**Affiliations:** Department of Biological Science, National Sun Yat-Sen University, Kaohsiung, Taiwan; Departments of Internal Medicine, Kaohsiung Chang Gung Memorial Hospital and Chang Gung University College of Medicine, Kaohsiung, Taiwan; Departments of Neurology, Kaohsiung Chang Gung Memorial Hospital and Chang Gung University College of Medicine, Kaohsiung, Taiwan; Departments of Emergency Medicine, Kaohsiung Chang Gung Memorial Hospital and Chang Gung University College of Medicine, Kaohsiung, Taiwan; Departments of Neurosurgery, and Radiology, Kaohsiung Chang Gung Memorial Hospital and Chang Gung University College of Medicine, Kaohsiung, Taiwan; Departments of Radiology, Kaohsiung Chang Gung Memorial Hospital and Chang Gung University College of Medicine, Kaohsiung, Taiwan; Department of Neurology, Chang Gung Memorial Hospital, 123, Ta Pei Road, Niao Sung Hsiang, Kaohsiung, Taiwan

**Keywords:** Caspase, Leukocyte apoptosis, Systemic lupus erythematosus, Interferon

## Abstract

**Background:**

This study aimed to explore the role of apoptosis initiators, caspase-9, caspase-10, mitochondrial anti-viral signaling protein (MAVS), and interferon regulatory factor 7 (pIRF7), in patients with systemic lupus erythematosus (SLE).

**Methods:**

Leukocyte apoptosis was determined by flow cytometry, including annexin V, APO2.7, and 7-amino-actinomycin D (7-AAD) on each subtype of leukocyte in 35 patients with SLE, 15 disease controls, and 17 volunteer normal controls. Levels of caspase-9, caspase-10, MAVS, and pIRF7 in mononuclear cells and the disease activity index (SLEDAI) in the SLE patients were determined. Correlation among intracellular adaptor proteins and caspase levels were calculated.

**Results:**

The SLE patients had higher APO2.7 in total leukocyte, lymphocyte, and monocytes, and higher late apoptosis markers in total leukocytes and neutrophils than normal controls (all *p* < 0.05). Disease activity was positively associated with the APO2.7 of CD19+ cells in SLE, but negatively associated with MAVS and caspase-9 levels (all *p* < 0.05). Markers of viral infection and anti-virus transcription factors like MDA5, MAVS, and pIRF7 were significantly higher in SLE patients than in disease controls (*p* < 0.05). Caspase-9 and caspase-10 levels positively correlated with MAVS and pIRF7 in SLE patients (*p* < 0.05).

**Conclusions:**

The disease activity of SLE is positively associated with APO2.7 level of CD19+ cells but negatively associated with MAVS and caspase-9 levels, which all point to a mitochondrial pathway.

## Introduction

Systemic lupus erythematosus (SLE) is a chronic systemic disease affecting mostly women of child-bearing age. It is the prototype of autoimmune diseases because of the variety of its proposed pathogenesis, including apoptosis. A previous study has demonstrated that lymphocyte apoptosis is increased and the removal of apoptotic cells is impaired in SLE. Apoptosis is also higher in active SLE compared to inactive SLE [1].

**Figure 1:**
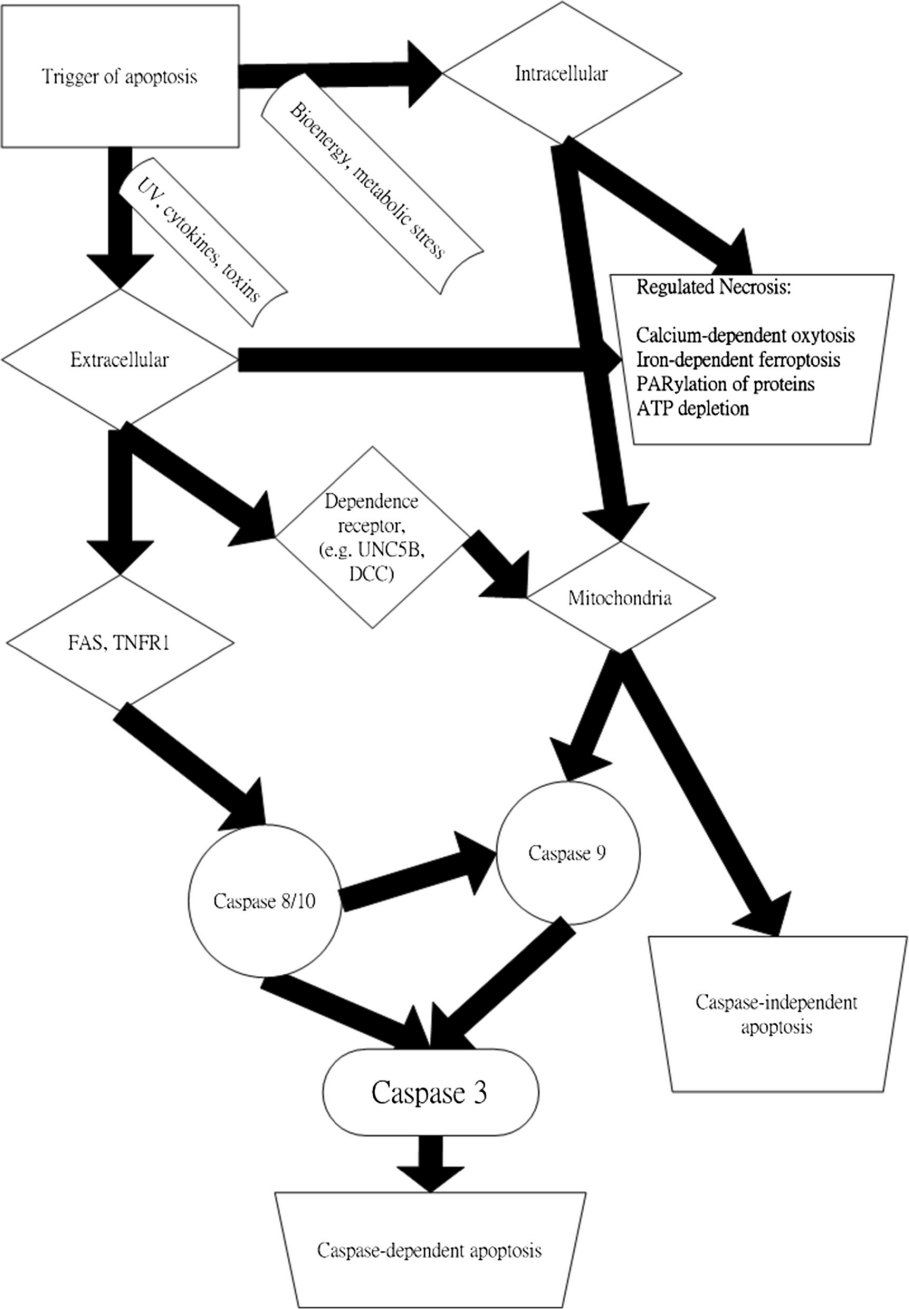
**Pathways of cell death and the interactions between initiator caspases.** This figure is mainly from the reference Cell Death Differ, 2012. 19(1): p. 107-20.

A recent study has demonstrated elevated apoptosis in SLE patients [2] and a correlation between disease activity and the apoptotic marker APO2.7 on CD19+ lymphocytes. Detection of APO2.7 apoptosis indicates that the inner side of the outer membrane of the mitochondria is turned over, which may be initiated by either an extrinsic or intrinsic apoptotic process [3]. The external apoptotic signal is executed through cell surface receptors (e.g. TNFR1, FAS), whereas caspase-10 is activated through proteolytic processes into several active isoforms (e.g. active cleaved form) [3]. Caspase-9 is known either as an initiator caspase of the mitochondrial intrinsic apoptotic pathway or an adaptor of the dependent receptor of apoptosis (Figure 1) [3,4].

By definition, peripheral blood mononuclear cells (PBMCs) include both lymphocytes and monocytes. In SLE patients, these two lineages of leukocytes are key players of the disease pathogenesis. The apoptotic signature may be started from the bone marrow of SLE patients [5]. Apoptosis and impaired apoptotic clearance are noted in SLE patients and such impaired mechanisms are documented as a pathogenesis of SLE [6]. The major functions of these two leukocyte lines are antigen presentation and the execution of adaptive immunity and interferon production against infection [7,8]. Aside from mononuclear cells of leukocytes, viruses have a role in inducing lupus and lupus flare-ups [9-12]. However, with the incorporation of the interferon pathway, the present study focuses on interferon regulatory factor 7 (IRF7) and its related protein in PBMCs, which have not been thoroughly investigated.

The post-viral immune response should induce IRF gene activation [13]. The change in IRF7 phosphorylation levels may be explained by the aberrant activation of the NLRP3 pathway [14], STAT1 pathway [15], or IRF3 [16] downstream of the mitochondrial anti-viral signaling protein (MAVS) due to inflammation. On the other hand, autoimmunity or the cytokine milieu in SLE may also be causes [17-19].

To date, little clinical research has focused specifically on the apoptosis pathway in SLE patients [20]. Because of the possible benefits in choice of appropriate immuno-suppressant regimens to reduce the degree of functional morbidity, there is a need to further improve the understanding of the pathogenesis of leukocyte apoptosis. Hence, this study explored the associations among caspases, phosphorylated IRF7 (pIRF7), and APO2.7 apoptosis in patients with SLE.

## Patients and methods

### Study patients

Thirty-five patients with definitive diagnosis of SLE and followed-up at the Rheumatology Out-patient Clinic for more than six months were prospectively evaluated. The diagnostic criteria for SLE were based on the 1997 revision of the 1982 American College of Rheumatology (ACR) classification criteria for SLE [21], while the clinical assessment of SLE disease activity was based on the SLE disease activity index (SLEDAI) [22]. All SLE patients were regularly followed-up at the clinics for more than six months to ensure that their conditions were stable and that their steroid dose and immune-modifying medication were not changed during the study period. For comparison, 18 age- and sex-matched healthy subjects were enrolled as healthy controls. Fifteen patients with a diagnosis of Sjogren’s syndrome, rheumatoid arthritis, vasculitis, or Behcet’s disease were also included as disease control patients.

The Institutional Review Committee on Human Research approved the study protocol and all of the participants provided informed consent. Patients were excluded if they had autoimmune diseases other than SLE or if they had fever or any infectious disorder that might affect the WBC count.

### Clinical assessments

All of the subjects underwent complete medical examinations upon enrollment. Complement levels and anti-double strand DNA levels were done regularly and collected at the same time upon enrollment. Biomarkers, including demographic data, complement levels, anti-ribosomal p autoantibody (a-rib p), and anti-double strand DNA autoantibody (a-dsDNA) levels, and disease activity index, were also collected. Since there were no clinical practice guidelines for SLE flare-up, the important decision in immuno-suppressant adjuvant treatment was whether or not to add a cytotoxic or corticosteroid-sparing drug like cyclophosphamide, methotrexate, azathioprine, mycophenolate mofetil, or leflunomide. The daily equivalent dose of steroids and the number of corticosteroid-sparing drugs used by SLE and disease control patients were recorded.

### Blood sampling and assessment of leukocyte apoptosis

Blood samples were collected by venipuncture of forearm veins of the SLE patients and controls.

### Flow cytometry assay for detecting apoptosis

All flow cytometry assays were performed within one hour after blood extraction to ensure that the results were as close as possible to an *in vivo* situation. Fixed amounts of blood were diluted 1:5 with PBS and 100 μL was stained with 10 μL of each of the following: fluorescence conjugated monoclonal antibodies against CD45-phycoerythrin (PE)-Cy5 (clone J33), CD61-fluorescein isothiocyanate (FITC; clone SZ21), and APO 2.7-PE (clone 2.7A6A3; Immunotech, Marseille, France), and titrated at saturating concentrations. The CD45-PE-Cy5 antibody reacted with the CD45 family of trans-membrane glycoproteins, which were expressed on the surface of all human leukocytes and were pan-leukocyte markers. The CD61-FITC antibody was a pan-platelet marker that reacted with the GPIIb/IIIa complex (fibrinogen receptor). The APO 2.7-PE antibody reacted with a 38-kDa mitochondrial membrane protein (7A6 antigen), which was detectable in non-permeabilized cells in the late apoptotic state [23].

Annexin V staining, relevant to early apoptosis, produced similar results but was rejected for questionable reliability under fixation conditions, with formaldehyde clearly biasing the staining results. Mouse immunoglobulin G-PE was a control for non-specific staining, which did not differ from the APO2.7-PE signal on platelets, such that each subject was used as its own control without changing the sample tube. After 30 minutes of incubation in the dark at room temperature, the stained samples were diluted with 0.5 ml of FACSFlow (Becton Dickinson, San Jose, CA).

Flow cytometry analysis was performed immediately after staining using an Epics XL flow cytometer (Beckman Coulter, Fullerton, Calif) and CellQuest software. Five thousand CD45-PE-Cy5+ cells per sample were acquired in a combined forward and side scatters and deep-red FL4 fluorescence (CD45-PE-Cy5) leukocyte gate. Another 5000 CD61-FITC + cells per sample were acquired in a combined forward and side scatters and green FL1 fluorescence (CD61-FITC) platelet gate to define a negative control threshold for the measurement of apoptosis. Each subject was his/her own control.

Membrane phosphatidyl-serine was detected by annexin-V using a commercially available kit (Boehringer Mannheim, Indianapolis, Ind). The PBS-washed leucocytes were incubated with annexin V-FITC and 7-amino-actinomycin D (7-AAD) for 15 min at room temperature according to the manufacturer’s guidelines. Samples were transferred to 5 mL polypropylene tubes, diluted with 900 μL Hanks’ balanced salt solution, and placed on ice before analysis by flow cytometry. The samples were analyzed using an Epics XL flow cytometer (Beckman Coulter, Fullerton, CA) and CellQuest software. Fifteen thousand events were counted per sample. Low-fluorescence debris was gated-out of the analysis. Leukocyte subtypes were identified according to their CD45 expression intensity and divided into neutrophils, monocytes, and lymphocytes.

Annexin V-FITC staining was identified in fluorescent-1 and 7-AAD staining in fluorescent-4. The cells were identified as follows: early apoptotic cells if they were positive for marker annexin V-FITC but negative for 7-AAD; late apoptotic cells if they were positive for annexin V-FITC and 7-AAD; dead cells if they were negative for annexin V-FITC but positive for 7-AAD; and viable cells if they were negative for annexin V-FITC and 7-AAD.

Fixed amounts of blood were diluted 1:5 with PBS, while 100 μL was stained with 10 μL of each of the following: fluorescence conjugated monoclonal antibodies against CD4-phycoerythrin (PE)-Cy5, CD19-fluorescein isothiocyanate (FITC), and CD8- phycoerythrin (PE). After each of the above staining, further staining was done with annexin V-FITC, 7-amino-actinomycin D (7-AAD), or APO 2.7-PE (clone 2.7A6A3; Immunotech, Marseille, France), with titration at saturating concentrations. The samples were then transferred to 5 mL polypropylene tubes, diluted with 900 μL Hanks’ balanced salt solution, and placed on ice before flow cytometry.

The samples were analyzed using an Epics XL flow cytometer (Beckman Coulter, Fullerton, Calif) and CellQuest software. Fifteen thousand events were counted per sample. Lymphocyte subtypes were identified according to their surface antigen (i.e., CD4+, CD8+, or CD19+) expression intensity. A database coordinator was responsible for monitoring all data collection and entry.

### Western blot analysis

Peripheral blood mononuclear cell (PBMC) intracellular protein levels of caspase-9, caspase-9c, caspase-10, caspase-10-c, and pIRF7 were detected by Western blot method. The PBMCs were separated by Ficoll-Paque (GE Healthcare Bio-Sciences AB, Stockholm, Sweden) density gradient centrifugation. The PBMC protein was extracted with RIPA Buffer containing 1 mmol/L protease inhibitor and 1 mmol/L phosphatase inhibitor. Proteins with sample buffer were then boiled for 10 min and aliquots of the supernatants (40 μg/lane) were fractionated by electrophoresis in 10% SDS acrylamide gel, electro-transferred to PVDF membranes, and blocked with 5% non-fat powdered milk in TBS-T (20 mmol/L Tris-base [pH 7.6], 137 mmol/L NaCl, 0.5% Tween-20).

The membranes were then incubated with commercially available rabbit polyclonal antibodies recognizing caspase-9 (Cell signaling, #9501), phospho-IRF-7 (Cell signaling, #5184), rat polyclonal antibodies recognizing caspase-10 (Biolegend, #645202) at 4°C overnight. Following extensive washing with TBS-T, the blots were incubated for 60 min with secondary antibodies. After brief rinsing with TBS-T, immuno-reactive protein bands were visualized by a chemiluminescence-based procedure using the ECL detection kit (Thermo, SuperSignal West Femto Maximum Sensitivity Substrate) according to the manufacturer’s instructions. The signal was quantified densitometrically by Quantity One 1-D analysis software (Bio-Rad USA, Life Science Research, Hercules, CA).

Activated caspase-9 was demonstrated by cleaved caspase-9 (active caspase-9, caspase-9c, 37 kd) from original caspase-9 (caspase-9, 47 kd) [18]. Activated caspase-10 was demonstrated by cleaved caspase-10 (active caspase-10, caspase-10c, 43 kd) from original caspase-10 (caspase-10, 59 kd) [19].

### Determination of intracellular protein concentration by enzyme linked-immuno-sorbent assay (ELISA)

Intracellular protein levels and serum levels of MDA5 were detected by commercial ELISA kit. Aliquots of the supernatants of protein (40 μg) were tested using a commercial ELISA kit to determine the concentration of intracellular MDA5 (APOtech, #APO-54 N-035, Epalinges, Switzerland). A commercial ELISA kit was used to determine the concentration of serum MDA5 (Cusabio, CSB-EL011017HU, China).

### Statistical analysis

Data were expressed as mean ± SD or median (inter-quartile range). Categorical variables were compared using Chi-square test or Fisher’s exact test, as appropriate. Continuous variables (i.e., surface markers of leukocytes and intracellular protein levels) were square root transformed to improve normality, and then compared between two groups using Student’s t-test and among three groups using one-way analysis of variance (ANOVA). Correlation analysis was used to explore the relationship between the mean steroid dosages and mean SLEDAI score, and variables such as surface markers of leukocytes, and intracellular protein levels. Statistical significance was set at *p* < 0.05. All statistical calculations were performed using the SAS software package, version 9.1 (2002, SAS Statistical Institute, North Carolina).

## Results

### Baseline characteristics of the study patients

The baseline characteristics, laboratory data, and apoptosis markers of the SLE patients, diseases controls, and healthy controls were listed in Table 1. The 15 diseases control patients included ten with Sjogren’s syndrome, two with rheumatoid arthritis, two with vasculitis, and one with Behcet’s disease. The clinical symptoms of the 35 SLE patients included neurologic involvement in 15, musculo-skeletal involvement in 10, hematologic involvement in seven, renal involvement in five, cardio-respiratory involvement in three, and muco-cutaneous involvement in three. Ten SLE patients had more than one organ involvement. There was no significant difference in age and sex among the three groups. The mean dosages of steroids and the number of immune-modifying medication were similar between the SLE and disease control groups (*p* > 0.05) (Table 1).

**Table 1:** **Demographic data of SLE patients and healthy controls**

	**Healthy controls (n = 18)**	**SLE patients (n = 35)**	**Disease control (n = 15)**	***p*** **value**
Age (y) (mean ± SD)	43.78 ± 10.90	40.24 ± 11.78	47.53 ± 11.06	0.17
Male/female	5/13	3/32	1/13	0.92
Peripheral blood studies				
Leukocytes (×1000/ml)	5.85 ± 2.15	6.35 ± 2.61	7.92 ± 3.11	0.12
% granulocyte	61.60 ± 12.76	67.89 ± 12.63	72.36 ± 16.25	0.25
% lymphocyte	31.01 ± 10.85	23.65 ± 10.87	20.46 ± 14.77	0.18
% monocyte	5.63 ± 1.74	6.14 ± 2.71	4.63 ± 2.10	0.48
Hemoglobin (mg/dL)	13.32 ± 1.77	11.73 ± 1.73	12.52 ± 1.58	<0.01*
Platelet counts (×10000/ml)	23.19 ± 6.82	22.19 ± 8.91	23.05 ± 8.60	0.93
c3 level (median, IQR)	ND	91.0 (61.4, 107.5)	ND	
c4 Level (median, IQR)	ND	19.30 (9.2, 24.)	ND	
a-dsDNA (median, IQR)	ND	13.4 (1.78, 67)	ND	
a-rib p (median, IQR)	ND	0.10 (0.1, 18)	ND	
SLEDAI score ( median, IQR)	ND	6 (4,12)	ND	
Medication used during at blood test				
Equivalent prednisolone dose per day (mg)		0 (0,8.75)	0 (0,8.75)	0.90
Immune modulators except steroid (number)		1 (1,1)	1 (1, 1.25)	0.48
Leukocyte Apoptosis by flow cytometry				
Leukocyte apoptosis (%)				
Annexin V (%)	10.7 (8.9, 13.8)	11.9 (8.6, 17.1)	12.1 (9.3, 20.6)	0.41
APO2.7 (%)	0.7 (0.5, 0.9)	1.3 (0.9, 2.0)	1.17 (0.9, 2.1)	<0.01*
Annexin V + 7AAD (%)	3.34 (2.85, 4.78)	6.34 (3.7, 9.7)	7.06 (5.16, 13.95)	<0.01*
Neutrophil apoptosis (%)				
Annexin V (%)	14.5 (10.0, 16.7)	19.4 (11.5, 29.8)	18.9 (14.4, 28.5)	0.08
APO2.7 (%)	0.51 (0.39, 0.62)	0.57 (0.41, 1.6)	0.82 (0.35, 1.39)	0.18
Annexin V + 7AAD (%)	3.7 (2.1, 7.6)	8.6 (2.7, 19.2)	15.94 (10.9, 31.4)	<0.01*
Lymphocyte apoptosis (%)				
Annexin V (%)	4.85 (4.01, 7.26)	6.28 (4.18, 9.52)	6.97 (4.00, 12.47)	0.16
APO2.7 (%)	0.34 (0.22, 0.45)	0.67 (0.4, 0.8)	0.79 (0.60, 1.01)	<0.01*
Annexin V + 7AAD (%)	1.76 (1.17, 2.40)	2.12 (1.31, 2.87)	2.20 (1.22, 3.54)	0.49
Monocyte apoptosis (%)				
Annexin V (%)	16.7 (13.0, 21.8)	16.4 (10.9, 22.1)	18.2(14.0, 23.9)	0.65
APO2.7 (%)	1.7 (1.05, 2.44)	2.8 (1.9, 5.2)	2.3 (1.3, 4.6)	0.02*
Annexin V + 7AAD (%)	5.5 (4.2, 6.6)	8.0 (4.3, 14.4)	8.2 (4.5, 17.0)	0.07

*Abbreviations*: SLE, systemic lupus erythematosus; IQR, inter-quartile range.

Data presented with median (IQR) or mean ± SD.

Continuous variables among three groups were compared using one-way ANOVA (analysis of variance) between healthy group, SLE, and diseases control.

Comparison between the three groups with non-normalized distributed parameters was calculated using the Kruskal-Wallis test, while post-hoc analysis was by Mann-Whitney U test.

*p < 0.05.

### Leukocyte apoptosis in patients with SLE and the controls

The laboratory data and percentage of leukocyte apoptosis among the three groups were also listed in Table 1. Except for hemoglobin level, all of the cell counts were not significantly different. Nonetheless, the SLE patients had higher apoptosis markers, including higher APO2.7 (*p* < 0.0001) and late apoptosis markers (*p* = 0.0019) in leukocytes. There were higher late apoptosis markers among neutrophils (*p* = 0.0479), higher APO2.7 apoptosis markers among monocytes (*p* = 0.0018), and higher APO2.7 apoptosis markers among lymphocytes (*p* = 0.0004) (Table 1).

### Correlation between clinical severity and percentage of monocytes and lymphocyte apoptosis

Correlation analysis was used to test the influence of leukocyte apoptosis on SLEDAI disease activity. The statistical results (correlation coefficient, *p* value) revealed that only the percentage of APO2.7 on CD19+ cells was positively associated with disease activity (r = 0.59, *p* = 0.01) (Table 2). The other subsets of leukocyte apoptosis were not correlated with SLEDAI disease activity. Further exploring the relationship between percentage of APO2.7 of CD19+ cell apoptosis and levels of autoantibodies revealed no correlation. Specifically, percentage of APO2.7 of CD19+ cell apoptosis did not correlate with the level of anti-Ro52 (r = 0.35, *p* = 0.39), anti-Ro60 (r = 0.29, *p* = 0.48), anti-La (r = 0.23, *p* = 0.34), anti-RNP (r = 0.01, *p* = 0.99), anti-Sm (r = 0.41, *p* = 0.11), anti-dsDNA (r = -0.28, *p* = 0.22), C3 (r = -0.16, *p* = 0.49), or C4 (r = 0.01, *p* = 0.98). Lastly, there was no significant relationship between the percentage of each apoptosis marker (e.g., Annexin V, APO2.7, annexin V + 7AAD) and steroid dosage in the SLE patients (all *p* > 0.05) (Table 2).

**Table 2:** **Correlation analysis between leukocyte apoptosis markers and disease activity and steroid dosage in patients with systemic lupus erythematosus**

	**SLE diseases activity**		**Steroid dosage**	
	**r**	**p**	**r**	**p**
CD4+ cell Annexin V (%)	0.02	0.93	0.01	0.96
CD4+ cell Annexin V + 7AAD (%)	-0.09	0.70	-0.33	0.12
CD8+ cell Annexin V (%)	-0.30	0.19	-0.17	0.45
CD8+ cell Annexin V + 7AAD (%)	-0.06	0.79	-0.19	0.39
CD19+ cell Annexin V (%)	0.17	0.46	-0.19	0.39
CD19+ cell Annexin V + 7AAD (%)	0.12	0.61	-0.15	0.50
Monocyte Annexin V (%)	0.01	0.97	-0.11	0.54
Monocyte Annexin V + 7AAD (%)	0.04	0.85	-0.14	0.46
APO2.7 CD4+ cell (%)	0.17	0.51	-0.29	0.20
APO2.7 CD8+ cell (%)	0.32	0.19	-0.12	0.61
APO2.7 CD19+ cell (%)	0.59	0.01*	0.17	0.46
APO2.7 monocyte (%)	0.05	0.77	-0.17	0.35

*Abbreviations*: r, correlation coefficient; p, p value; SLEDAI, Systemic lupus erythematosus disease activity index 2000.

*p < 0.05.

### Correlation between each intracellular protein level and disease activity in SLE patients

**Figure 2:**
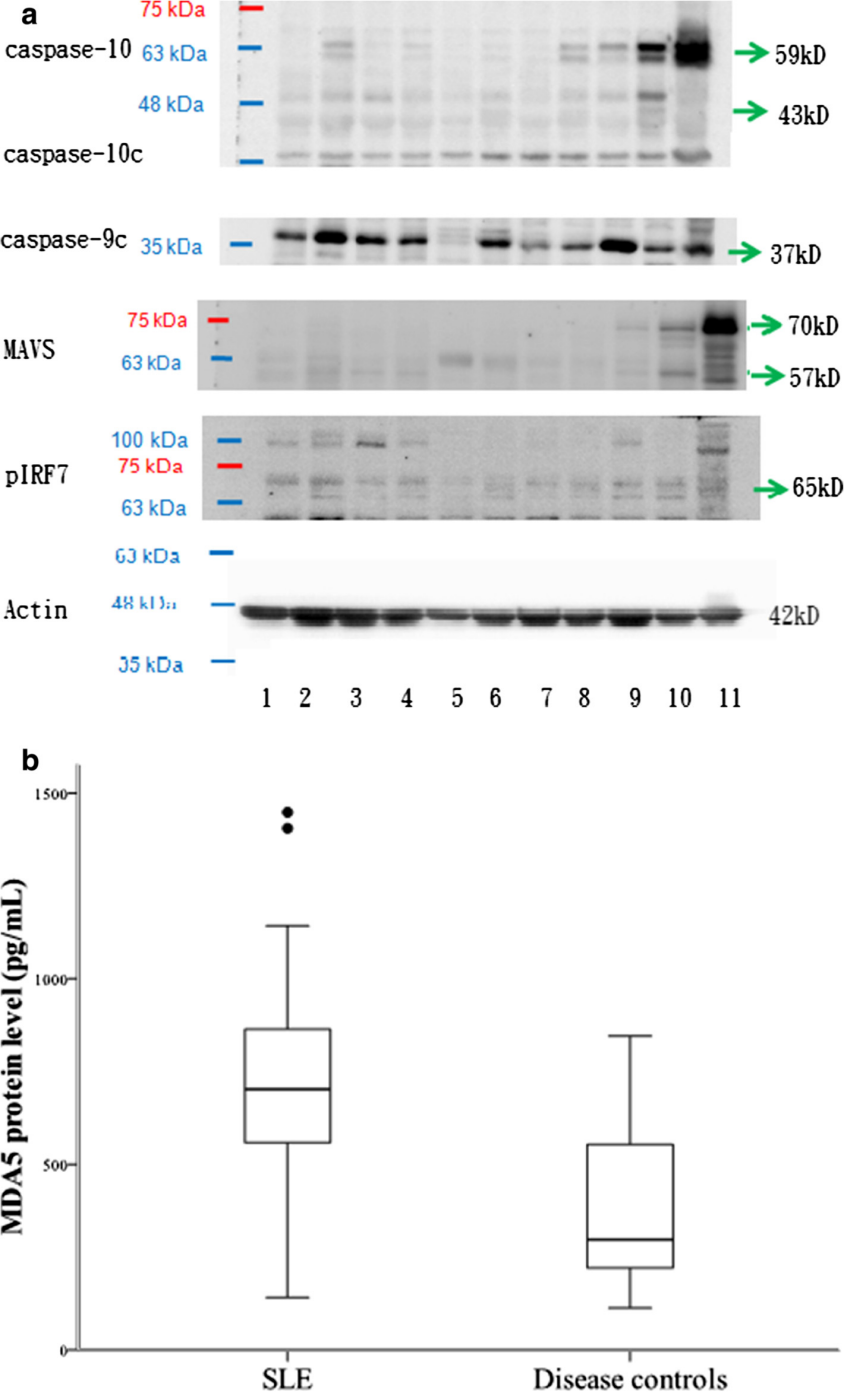
**Intracellular protein concentration from peripheral blood mononuclear cells in patients with systemic lupus erythematosus (SLE). (a)** Lanes 1-7: SLE patients; Lane 8-10: disease control patients; and Lane 11: Jurkat T cell lysate. **(b)** MDA5 levels, detected by ELISA method, were significantly different between SLE patients and disease controls.

The intracellular proteins, including active cleaved caspase 10c (43 kD), caspase 9c (37 kD), active MAVS (57 kD), and pIRF7 (65 kD) were determined by Western blot method (Figure 2a), while MDA5 levels were determined by ELISA (Figure 2b). The correlation between each intracellular protein, including MDA5, active cleaved caspase-9, active cleaved caspase-10, pIRF7, and active MAVS revealed that active cleaved caspase-9 and active MAVS level both negatively correlated with SLEDAI (*p* = 0.01 and *p* < 0.05, respectively) (Table 3).

**Table 3:** **Correlations analysis between activated caspase-9, activated caspase-10, pIRF7, virus infection markers, and systemic lupus erythematosus disease activity**

		**SLEDAI**	**activated caspase 9**	**activated caspase 10**	**pIRF7**	**activated MAVS**	**MDA5**
SLEDAI	r	1.00	-0.47^*^	-0.31	-0.31	-0.36^*^	-0.03
	p	X	0.01^*^	0.13	0.09	<0.05^*^	0.90
activated caspase 9	r		1.00	0.81^*^	0.83^*^	0.88^*^	0.39
	p		X	<0.01^*^	<0.01^*^	<0.01^*^	0.06
activated caspase 10	r			1.00	0.91^*^	0.85^*^	0.23
	p			X	<0.01^*^	<0.01^*^	0.33
pIRF7	r				1.00	0.84^*^	0.14
	p				X	<0.01^*^	0.51
activated MAVS	r					1.00	0.27
	p					X	0.21
MDA5	r						1.00
	p						X

*Abbreviations*: r, correlation coefficient; p, p value; SLEDAI, Systemic lupus erythematosus disease activity index 2000; pIRF7, phosphorylated interferon regulator factor 7; MAVS, mitochondrial antiviral signaling protein; MDA5, Melanoma Differentiation-Associated protein 5.

*p < 0.05.

Active cleaved caspase-9 positively correlated with active cleaved caspase-10 (*p* < 0.01). Both the active cleaved caspase-9 and caspase-10 positively correlated with pIRF7 (*p* < 0.01). Despite the absence of correlation between MDA5 level and other molecules, activated MAVS positively correlated with the active cleaved caspase-9, caspase-10, and pIRF7 (all *p* < 0.01).

### Correlation between original and cleaved active caspase-9 and -10 in SLE

**Figure 3:**
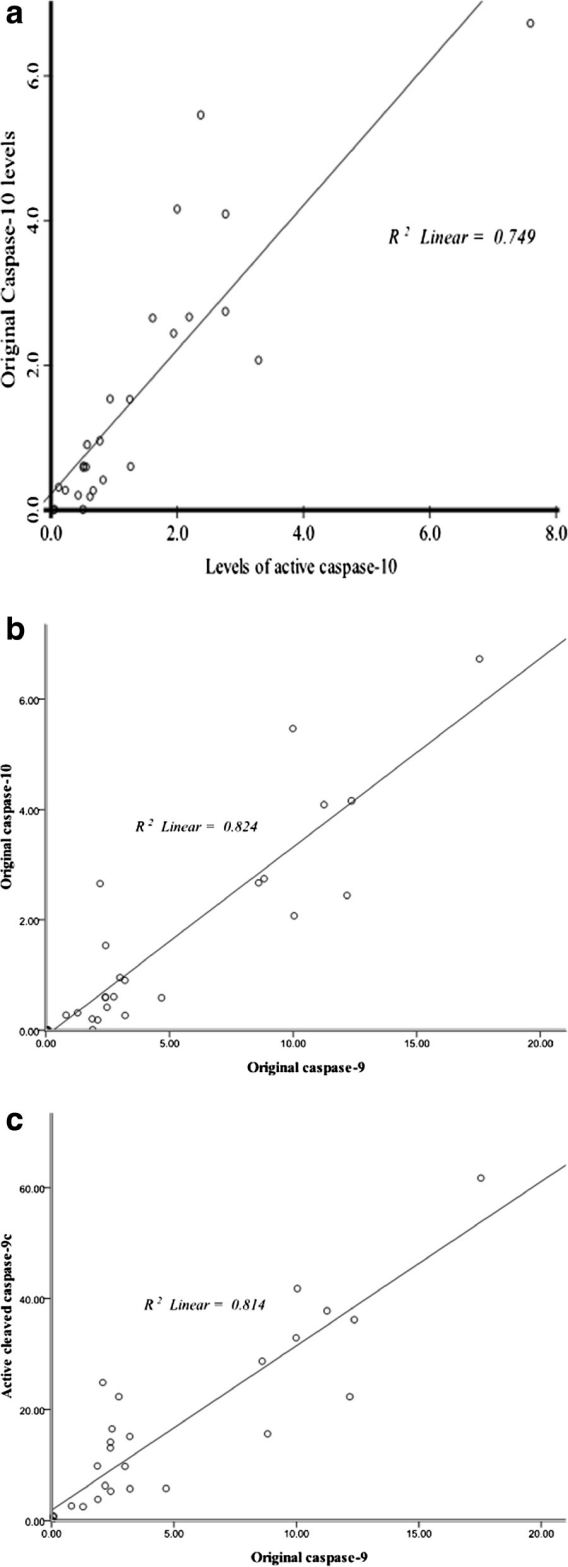
**Correlations between original and cleaved caspase-9 and caspase-10 in systemic lupus erythematosus patients. (a)** In PBMCs, original caspase-10 was positively associated with active cleaved casepase-10c (*p* < 0.05). **(b)** Original caspase-10 was positively associated with original caspase-9 (*p* < 0.05). **(c)** Original caspase-9 was positively associated with active cleaved casepase-9 (*p* < 0.05). Abbreviations: r, correlation coefficient.

In PBMCs, caspase-10 was positively associated with caspase-10c (*p* < 0.001) (Figure 3a). Caspase-9 and caspase-10 positively correlated with each other (*p* < 0.001) (Figure 3b). Caspase-9 positively associated with active cleaved casepase-9c (*p* < 0.001) (Figure 3c).

### Correlation between disease activity and MAVS protein level in SLE

**Figure 4:**
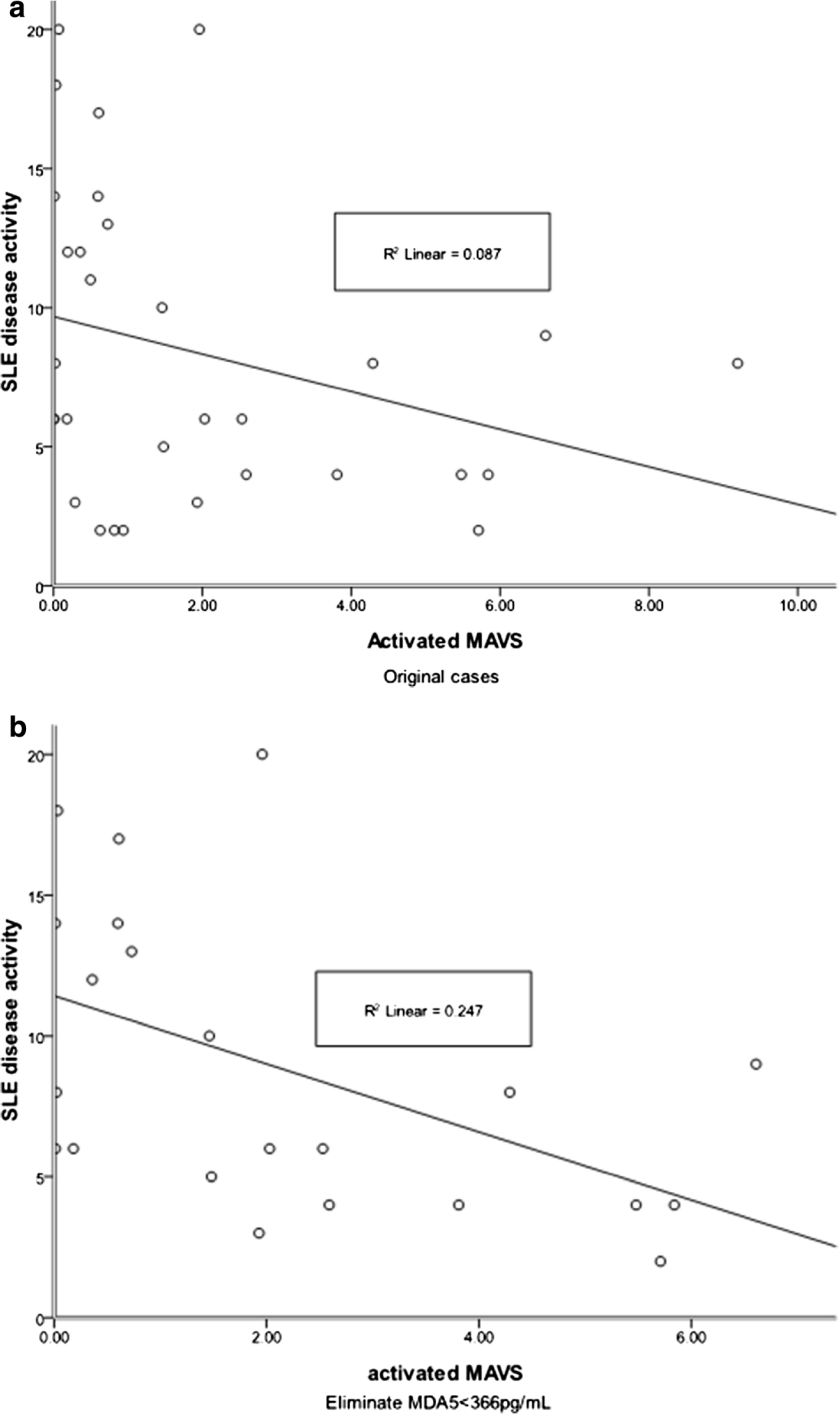
**Correlation between MAVS and SLEDAI. (a)** Origin correlation between MAVS and SLEDAI. **(b)** Correlation between MAVS and SLEDAI after eliminating the low-MDA5 sub-group.

The negative coefficient between MAVS and SLE disease activity (Table 3) was enhanced if patients with lower MDA5 levels (cut-off value, 366 pg/mL, mean of disease controls) were removed (Figure 4). The *p* value changed from 0.04955 to 0.01123, indicating that SLE was a heterogeneous group and could be divided into at least two subgroups according to MDA5 levels. Disease activity negatively correlated with MAVS in the high MDA5 level SLE patients (*p* < 0.05) but not in the low MDA5 level SLE patients (*p* = 0.67) (data not shown).

## Discussion

The present study has several major findings. First, there are comparable apoptosis markers between SLE and disease control patients, even though only neutrophil late apoptosis is statistically significantly higher in disease controls than in SLE patients (*p* < 0.05) (Table 1). Second, SLE disease activity is positively associated with CD19+ cell APO 2.7 marker level (Table 2), but negatively associated with MAVS and caspase-9 levels (Table 3) (*p* < 0.05). Third, although most of the surface markers of apoptosis are not significantly different between SLE and disease controls, the levels of intracellular molecules are significantly different (*p* < 0.05) (Table 4). Fifth, markers of viral infection and anti-virus transcription factors, including MDA5, MAVS, and pIRF7, are significantly higher in SLE patients than in disease controls (*p* < 0.05) (Table 4). Sixth, caspase-9 and caspase-10 levels are positively correlated with anti-virus factors like MAVS and pIRF7 (*p* < 0.05) (Table 3), but not MDA5 levels. Lastly, SLE disease activity is more negatively associated with MAVS among patients with high MDA5 levels (Figure 3).

**Table 4:** **Comparison of intracellular molecules between patients with systemic lupus erythematosus and disease controls**

	**SLE**	**Disease controls**	
	**n = 31**	**n = 15**	***p*** **value**
Intracellular protein levels			
MDA5 (pg/mL)	701.53 (543.45,885.96)	296.74 (216.50,567.40)	<0.01*
Activated caspase 9	9.74 (0.81, 22.34)	2.20 (0.26, 3.58)	0.06
Activated caspase 10	0.77 (0.51, 2.09)	0.15 (0.04, 0.24)	<0.01*
pIRF7	0.10 (0.04, 0.30)	0.03 (0.01, 0.04)	<0.01*
Activated MAVS	0.82 (0.19, 2.59)	0.24 (0.11, 0.42)	0.02*

Data presented with median, IQR; IQR, inter-quartile range.

The levels of pIRF7, activated caspase 9, caspase 10, and MAVS from Western blot method were normalized with actin levels.

*Abbreviation:* pIRF7, phosphorylated interferon regulator factor 7; MAVS, mitochondrial anti-viral signaling protein.

*p < 0.05.

This present study confirms that pronounced apoptosis in SLE and disease activity is positively associated with APO2.7 of CD19+ cells [2]. Disease activity is also negatively associated with caspase-9 and MAVS in PBMCs (Table 3). These all point to a mitochondrial pathway. The explanation may be that when disease activity increases, the PBMCs are activated and anti-apoptotic molecules accumulate on the mitochondrial membrane [24-27], which suppress caspase-9. Moreover, another article mentions that the expression of caspase 9 is decreased in female SLE patients [20]. Active cleaved caspase-9 may come from any lineage of PBMCs, such as activation-induced-cell-death of γδ-T cell, which depends on caspase-9 [28], or through pathways not dependent on caspase in other CD4+ T cells [29].

During disease flare-up, CD19+ cells are prone to producing more autoantibodies with mitochondrial activation, which may lead to APO2.7-related apoptosis. However, the detailed apoptosis mechanism remains unknown. On the other hand, the reason that caspase-10 is not correlated with disease activity may be that there are too many confounding factors affecting the extrinsic apoptosis pathway, like TNF-α [30] and other stress signaling [31].

The negative correlation between disease activity and MAVS is contrary to a previous clinical observation [9]. Thus, the role of MDA5 in this correlation has been investigated by determining the levels of MDA5 among SLE and disease control patients. A previous study comparing MDA5 levels between SLE and healthy controls [32] shows the MDA5 is significantly higher in SLE than in healthy patients. In the present study, MDA5 levels, as well as the anti-virus signaling proteins MAVS and pIRF7, are significantly higher in SLE than in disease control patients (Table 4). This indicates that SLE patients are more vulnerable to recurrent viral infection than disease controls.

Furthermore, using the mean MDA5 level from disease controls as reference, disease activity is negatively correlated to MAVS in SLE patients with high MDA5 levels (*p* < 0.05), but not in those with low MDA5 levels (*p* = 0.67) (Figure 4). This indicates that MAVS is only negatively associated with disease activity in a subset of SLE patients. Further studies to determine the specific role of MDA5 and MAVS in SLE are needed.

The three major IRFs (i.e., IRF3, IRF5, and IRF7) are downstream of mitochondrial signaling to promote interferon-stimulated gene expression [33,34]. According to a previous study, SLE is an interferon-associated disease. Thus, this study focused on interferon regulating factors, particularly on IRF7 because of a previous study by Becker et al. [31], wherein IRF7 is the only one significantly different between healthy and SLE CD19+ cells. In the current study, SLE CD19+ cells are associated with disease activity in SLE patients.

Caspase-9 is mainly an intracellular initiator caspase and is related to extrinsic apoptosis from dependent receptors [3]. In the current study, caspase-9c is positively associated with MAVS and pIRF7 (Table 3). It will be interesting to elucidate the relationship between viral infection and caspase-9 activation, since virus-related cellular apoptosis has been demonstrated in some HIV infected patients [35], while caspase-9 activation is noted in chronic hepatitis C viral infection [36]. Caspase-9c and MAVS are both negatively associated with disease activity in this study (Table 3), which may hint that activation-induced-cell-death through mitochondrial pathway is different from the virus-related apoptosis and/or activation pathway in SLE patients. Virus could lead to IRF7 down-regulation [37] or activation [13,38,39]. Virus-related activation and activation-induced apoptosis of mononuclear cells in SLE through the mitochondrial pathway may exist but warrants further study.

There are several positive correlations between caspase-10c and MAVS, as well as caspase-10c and pIRF7 (Table 3). These indicate that caspase-10, like caspase-9, may be another major initiator of the caspase pathway in PBMCs in SLE. Caspase-10c is produced mainly after caspase-10 is activated and cleaved. Its molecular weight is around 43 kD [40]. Both the original caspase-10 (59 kD) and caspase-10c (43 kD) have the large protease subunit p20, recognized by the secondary antibody in Western blotting. Both molecules are functionally active [40].

Nonetheless, this study has several limitations. First, theoretically, it would have been better if PBMCs were divided into different cell lineages. In this study, as in other studies, PBMCs are used as a surrogate rather than lymphocytes only because of the technical difficulty to differentiate lymphocytes from monocytes using flow cytometry study [41,42]. Because lymphocytes account for the majority of PMBCs, the results are not much different from the true state. Second, this is a cross-sectional observational study. Theoretically, immuno-suppressant drugs can affect leukocyte activity and autoantibody levels even if SLE patients are in the convalescent stage. Lastly, the case number in this study is small. Large-scale prospective and longitudinal studies are needed to evaluate the prognostic contribution of leukocyte apoptosis on clinical outcome.

### Ethical approval

The study was approved by Chang Gung Memorial Hospital’s Institutional Review Committee on Human Research.
